# Advanced applications of DNA nanostructures dominated by DNA origami in antitumor drug delivery

**DOI:** 10.3389/fmolb.2023.1239952

**Published:** 2023-08-07

**Authors:** Yiming Zhang, Xinchen Tian, Zijian Wang, Haochen Wang, Fen Liu, Qipeng Long, Shulong Jiang

**Affiliations:** ^1^ Clinical Medical Laboratory Center, Jining First People’s Hospital, Shandong First Medical University, Jining, Shandong, China; ^2^ College of Traditional Chinese Medicine, Shandong University of Traditional Chinese Medicine, Jinan, Shandong, China

**Keywords:** DNA origami, DNA nanostructures, anti-tumor drug delivery, cancer treatment, DNA self-assembly technique

## Abstract

DNA origami is a cutting-edge DNA self-assembly technique that neatly folds DNA strands and creates specific structures based on the complementary base pairing principle. These innovative DNA origami nanostructures provide numerous benefits, including lower biotoxicity, increased stability, and superior adaptability, making them an excellent choice for transporting anti-tumor agents. Furthermore, they can considerably reduce side effects and improve therapy success by offering precise, targeted, and multifunctional drug delivery system. This comprehensive review looks into the principles and design strategies of DNA origami, providing valuable insights into this technology’s latest research achievements and development trends in the field of anti-tumor drug delivery. Additionally, we review the key function and major benefits of DNA origami in cancer treatment, some of these approaches also involve aspects related to DNA tetrahedra, aiming to provide novel ideas and effective solutions to address drug delivery challenges in cancer therapy.

## 1 Introduction

DNA origami is a new nanotechnology that utilizes numerous techniques to precisely fold DNA molecules into a variety of forms, yielding unique DNA nanostructures with enhanced properties. These structures have extensive applications in biomedical sciences, particularly in drug delivery. Cancer is a disease that develops when, in response to specific factors, normal cells undergo certain transformations that allow them to proliferate without limit and form malignant tumors. The biological mechanisms involved are complex, and malignancy is severe, with a poor prognosis. Currently, there are several types of anti-tumor medications available, including chemotherapy drugs, nucleic acid drugs, and protein drugs. Chemotherapy drugs, which are potent medications, indiscriminately attack both normal and cancerous cells within the body, frequently causing side effects such as bone marrow suppression, cardiac toxicity, and serious digestive and skin rea ctions, all of which significantly harm the patient’s health. While nucleic acid and protein-based biological preparations typically have low cytotoxicity, they are unstable within the body and can quickly be decomposed by endogenous hydrolytic enzymes, as well as potential immunogenicity, limiting their efficacy. Since DNA origami nanostructures (DONs) possess excellent editability, biocompatibility, and *in vivo* stability, they can be utilized to create DNA nanostructures with a variety of activities and anti-tumor medications. This enables drugs to access cancer cells with greater stability and precision, boosting treatment efficacy and lowering toxicity. Consequently, studying ways to employ DNA origami to address drug delivery issues has become a hot spot in medical research. This review will comprehensively analyze the most recent research advances and development trends of DNA origami in delivering anti-tumor drugs, exploring its role and benefits in cancer treatment, with the aim of providing new ideas and directions for addressing drug delivery issues in tumor therapy more effectively.

## 2 DNA origami for drug delivery design

### 2.1 Overview of the DNA origami principle

A DNA molecule is a polymer consisting of two continuous single strands of deoxyribonucleotides that run parallel and in opposite directions. Each strand contains four types of bases, namely, adenine (A), guanine (G), thymine (T), and cytosine (C). The nucleotides combine to complementary pairs A-T and C-G, creating stable links between the strands and giving foundation to DNA’s double helix shape. Because of the selectivity and stability of base pairing, DNA molecules may be folded and assembled, which can then be manipulated by changing the sequence arrangement and template shape to construct complex three-dimensional structures. Using this DNA base pairing theory, professor Rothemund from the California Institute of Technology introduced the concept of DNA origami in 2006 (Rothemund, 2006). By designing and arranging DNA sequences, he successfully created artificial shapes such as triangles and stars. DNA origami is a unique bottom-up approach to constructing nanoscale structures made from DNA in a self-assembling manner. Through nucleic acid sequence hybridization, a long single-stranded DNA, typically the genome of M13 phage, is used as a scaffold, and hundreds of short single-stranded DNAs, or staple strands, are used to form pre-designed nanostructures with specific sizes and shapes. The short DNA strands act like staples on a bookbind, crossing multiple binding domains on the DNA scaffold to create the appropriate 2D or 3D structure. This causes the scaffold folding and twisting from its initial linear configuration into the desired nano-pattern. Finally, the short and long DNA strands are heated in an alkaline solution (Typically, it is a buffer solution consisting of tris acetate buffer with a pH of around 8.), automatically and stably binding to form the initially designed structures. DNA origami presents a promising technique for creating intricate, functional nanostructures for applications in various fields, including medicine and electronics (Jiang et al., 2019; Dey et al., 2021).

**FIGURE 1:**
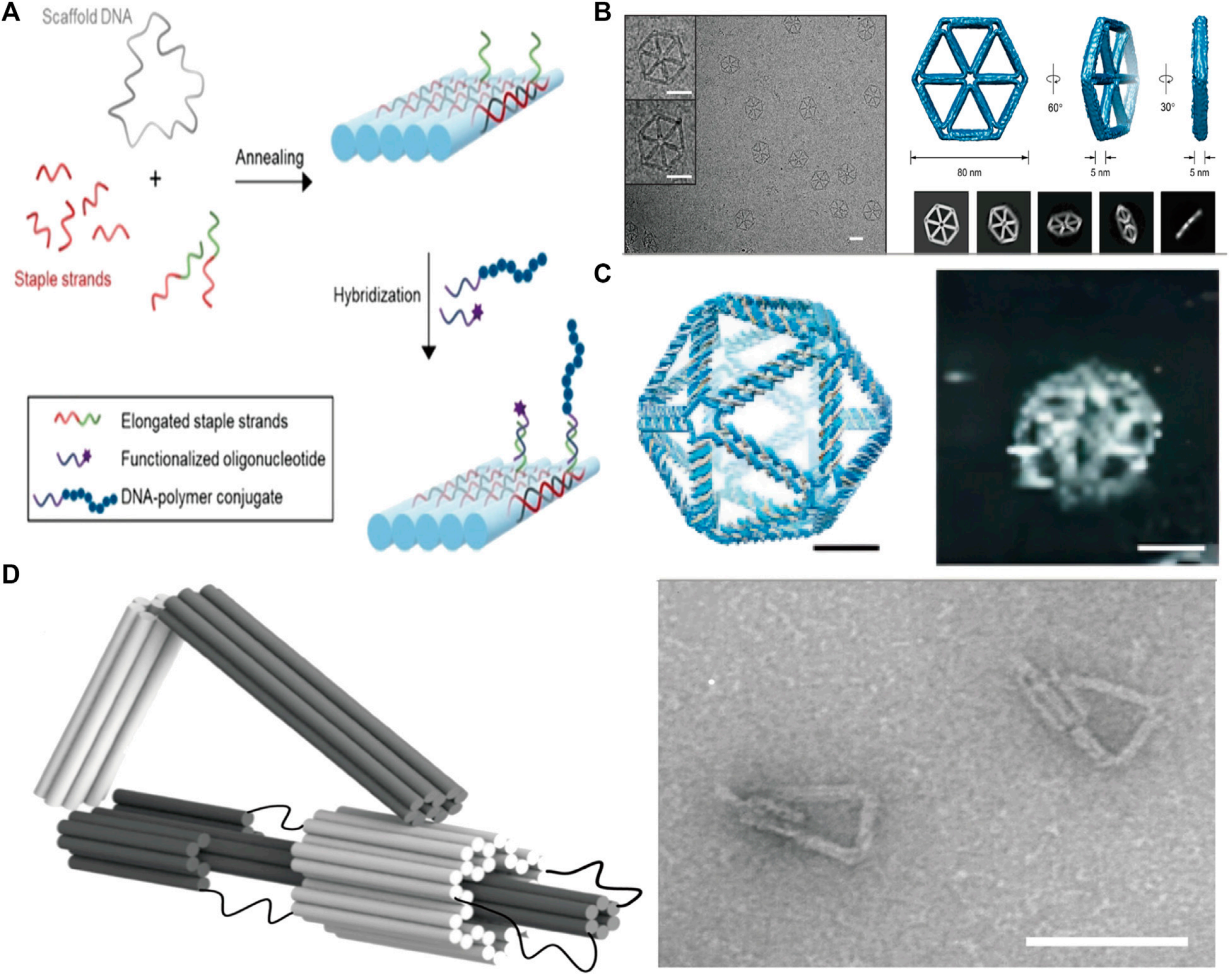
**(A)** Schematic diagram of the principle of DNA origami. Reproduced with permission (Hannewald et al., 2021) Copyright © 2021, John Wiley and Sons. **(B)** 2D wireframe hexagonal DNA nanostructure made using DNA origami. Reproduced with permission (Wang et al., 2022b) under copyright Creative Commons Attribution 4.0 International License (CC-BY license) **(C)** 3D wireframe DNA nanostructures in the shape of an icosahedron are formed using DNA origami techniques. Reproduced with permission (Veneziano et al., 2016) Copyright © 2016, The American Association for the Advancement of Science **(D)** A DNA nano-component with flexible motion capability made using DNA origami. Reproduced with permission (Marras et al., 2015) under copyright Creative Commons Attribution 4.0 International License (CC-BY license).

To produce a DONs, the initial step usually involves designing the sequence via programmatic software, such ascaDNAno (Douglas et al., 2009), ATHENA (Hyungmin et al., 2020), and Adenita (de Llano et al., 2020), widely utilized nowadays. Once the template strand and auxiliary folding strands are designed, they are mixed in a specific ratio for annealing, followed by subsequent functional modifications and purification of the resulting structure. Over the last 2 decades, scientists have created an array of 2D or 3D DNA folding structures (Figure 1).

DNA origami technology is programmable, allowing for the design and manufacturing of DNA nanostructures with features and functions that may be tuned to individual requirements. Drug delivery, sensors, and nanocircuits are just a few examples of the many applications that could benefit greatly from such structures.

### 2.2 Strategies to construct DONs for drug delivery

Loading drugs onto DONs is a critical step in optimizing drug delivery. The stable structure and specific targeting properties of DONs make them ideal nanocarriers for efficient drug transportation. To load drugs onto DONs, an appropriate method must be selected based on the drug’s characteristics. This can be accomplished by the use of techniques like as intercalation, ligand recruitment, chain modification, hybridization, or encapsulation (Madhanagopal et al., 2018). For instance, the binding of doxorubicin (DOX) to either the G-C base pairs or grooves of the A-T rich regions in the double-stranded DNA can be used to combine DOX with DONs and generate a stable complex (Perez-Arnaiz et al., 2014). For larger-molecule drugs, their corresponding ligands can be chemically linked to DONs to achieve loading functionality. Alternatively, drugs can be covalently connected to component DNA chains and assembled as part of the structure (Liu et al., 2012; Chen et al., 2016; Schaffert et al., 2016). For nucleic acid drugs like cytosine-phosphorothioate-guanine (CpG) and small interfering RNA (siRNA), adding a complementary single-strand extension onto the DONs is suitable (Perez-Arnaiz et al., 2014). In general, selecting the appropriate drug-loading method is crucial in harnessing the full potential of DONs as a nanocarrier for optimal drug delivery results.

Enhancing drug delivery targeting is a critical strategy for developing efficient DON-based drug delivery systems. Traditional anti-tumor drugs, such as chemotherapy agents, frequently induce significant toxic side effects by attacking both malignant and healthy cells. However, designing and fabricating nanocarriers with specific targeting can release drugs only when necessary, reducing the harm to healthy cells. Special DONs can considerably improve targeting efficiency. Rectangular, triangular, or tubular-shaped DONs have shown a preference for accumulating in the kidneys. In particular, rectangular DONs have demonstrated a stronger kidney-protective effect similar to N-acetyl cysteine, which assists in clearing reactive oxygen species (ROS) from the kidneys (Jiang et al., 2018; Wang, 2019). The precise arrangement of interleukin-33 (IL-33) nanoarrays on rectangular DONs enables preferential delivery of IL-33 to the kidneys. Nano-rafts loaded with IL-33 exhibit a preference for accumulating in the kidneys, where they can stay for up to 48 h and continuously release IL-33. This leads to a rapid increase of type 2 innate lymphoid cells (ILC-2) and regulatory T cells (Tregs), facilitating the alleviation of ischemic acute kidney injury (AKI) (Li et al., 2021). Moreover, anchoring cell-specific antibodies, aptamers, peptides, ligands, or receptor-specific proteins on the surface of DONs can enable drug-loaded nanostructures to reach specific regions. For instance, triangular DONs modified with folate have been shown to enhance the targeting of M1 macrophages while helping the transition of pro-inflammatory M1 macrophages to anti-inflammatory M2 macrophages. This results in a reduction of reactive oxygen species (ROS) and nitric oxide (NO) levels, thereby promoting a better anti-inflammatory response (Ma et al., 2022b).

Improving drug stability is crucial for new biologics used against tumors, such as nucleic acid drugs, which are highly susceptible to hydrolytic enzymes, leading to poor stability *in vivo*. However, DONs as a shell can provide protection against environmental and physiological factors, boosting drug stability and prolonging its lifespan. For example, Lacroix et. alcombining human serum albumin with DONs can slow down the degradation rate of DNA structures *in vivo*, helping to stabilize siRNAs’ efficacy in cells (Lacroix et al., 2017). Ponnuswamy et al. employed oligolysine conjugated to PEG to shield DNA nanostructures, thus mitigating nucleolytic degradation (Ponnuswamy et al., 2017). Agarwal et al. utilized cationic poly (ethylene glycol)-polylysine block copolymer micelles as a robust and cost-effective strategy for safeguarding DONs (Agarwal et al., 2017). Gerling et al. enhanced the resistance of DNA origami structures against nucleases by inducing thymine dimer formation through ultraviolet irradiation. Some studies have indicated that encapsulating DNA origami within protective silica shells via sol-gel chemistry contributes to the stability of DONs (Liu et al., 2018b; Nguyen et al., 2019; Wassermann et al., 2023). Similarly, it has been suggested that mineralization of DONs surfaces with calcium phosphate enhances resistance against degradation by nuclease (Wu et al., 2021a).

**FIGURE 2:**
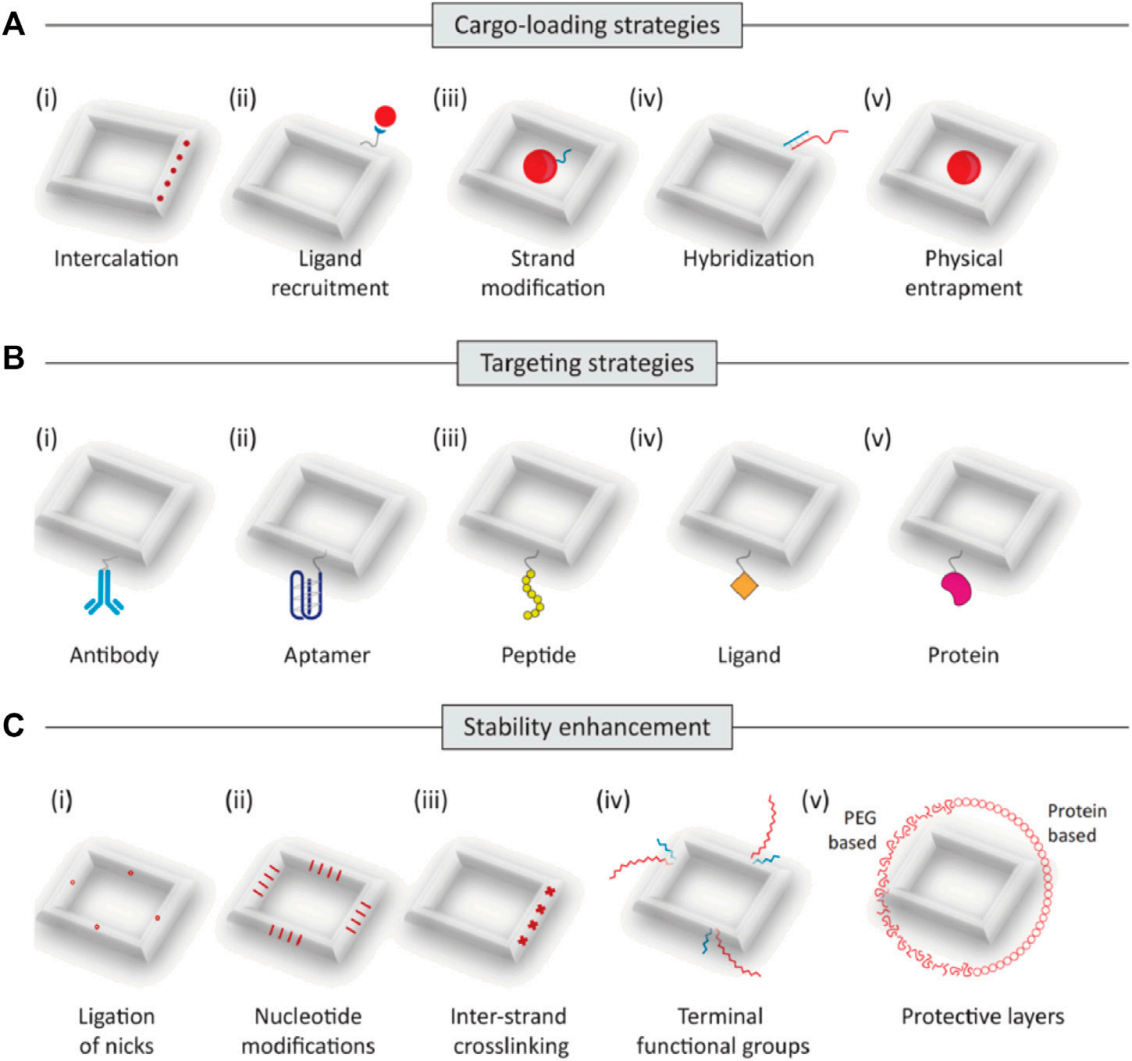
Trategies to construct DONs for drug delivery. **(A)** The main strategies of drug loading for DONs(as shown in i-v): intercalation, ligand recruitment, trand modification, hybridization, and entrapment. **(B)** Targeting strategies. Drug-loaded DONs can be designed to reach specified locations by using cell-specific antibodies, aptamers, peptides, ligands, or receptor-specific proteins (as shown in i-v). **(C)** Strategies to improve biostability. Modifications to improve the stability of DNA nanostructures include ligation of nicks, (ii) nucleotide modifications, (iii)inter-strand crosslinking, (iv) functional groups such as hexaethylene glycol and hexane diol, and (v) polyethylene glycol (PEG)-based or protein-based protective layers.

Moreover, some studies have shown that packaging proteins and peptides on the surface of DONs (Cho et al., 2014; Auvinen et al., 2017; Wang et al., 2020; Seitz et al., 2022) and increasing cross-linking quantity (Xin et al., 2022) can effectively prevent nucleases from degrading it, hence increasing nuclease resistance, regulating drug release rate, and improving DONs stability. Additionally, as shown by the findings of Rajendran et al., reinforcing linkages can enhance the thermal stability of DONs, which is important for maintaining stable release of thermosensitive anti-tumor drugs *in vivo* (Rajendran et al., 2021) (Figure 2).

## 3 Anti-tumor drug delivery dased on DNA origami

### 3.1 Targeted drug delivery

Using the DNA origami technique to construct nanostructures can also endow them with more functions beyond basic drug delivery. For instance, several medications, such as a combination of anti-tumor medications and adjuvants or different types of anti-tumor drugs, can be loaded onto DONs, including, to achieve comprehensive treatment and improve therapeutic efficacy while reducing side effects. Furthermore, employing DONs to load multi-functional nanocarriers can simultaneously support drug delivery, imaging, and therapy, which has significant implications for improving treatment outcomes (Lu et al., 2019; Zeng et al., 2021).

Targeted therapy is a sort of treatment that uses medications or other substances to identify and attack specific types of cancer cells while causing minimal damage to normal cells. Clinical practice has revealed that many drugs often suffer from shortcomings, such as multiple target points, high cell toxicity, and poor *in vivo* stability, which frequently lead to various side effects and poor therapeutic outcomes (Li et al., 2022). However, with the construction of suitable DNA nanostructures using DNA origami, drugs can be loaded onto the DNA folding vehicle. This reduces *in vivo* loss, delivers precisely to target cells, accurately kills specific targets without hurting normal cells, and diminishes toxic side effects while enhancing efficacy. Many studies have shown that DNA origami particles loaded with drugs can achieve targeted drug delivery, particularly for chemotherapeutic drugs, nucleic acid drugs, protein, and peptide drugs (Udomprasert and Kangsamaksin, 2017; Zhang et al., 2023).

DNA origami allows DNA molecules to be folded into various nanoscale structures, such as nanotubes and nanoboxes, which has notable benefits for targeted drug delivery. For instance, these nanoscale structures are highly controllable. By adjusting the folding sequence and length, the shape and size of the nanostructure can be precisely controlled, enabling accurate targeting and delivery of drugs (Yan et al., 2015). Additionally, DNA origami exhibits excellent selectivity and can target specific cells or tissues by designing DNA sequences with specific affinities, thus allowing for precise targeting of drugs (Li et al., 2013). Furthermore, DNA origami has a high drug-carrying capacity, efficiently encapsulating and releasing multiple types of drugs by adjusting spatial structure and size. Finally, using naturally occurring DNA molecules in the body results in extremely biocompatible nanostructures, reducing adverse impacts on the human body (Yan et al., 2015; Kumar et al., 2016).

**FIGURE 3:**
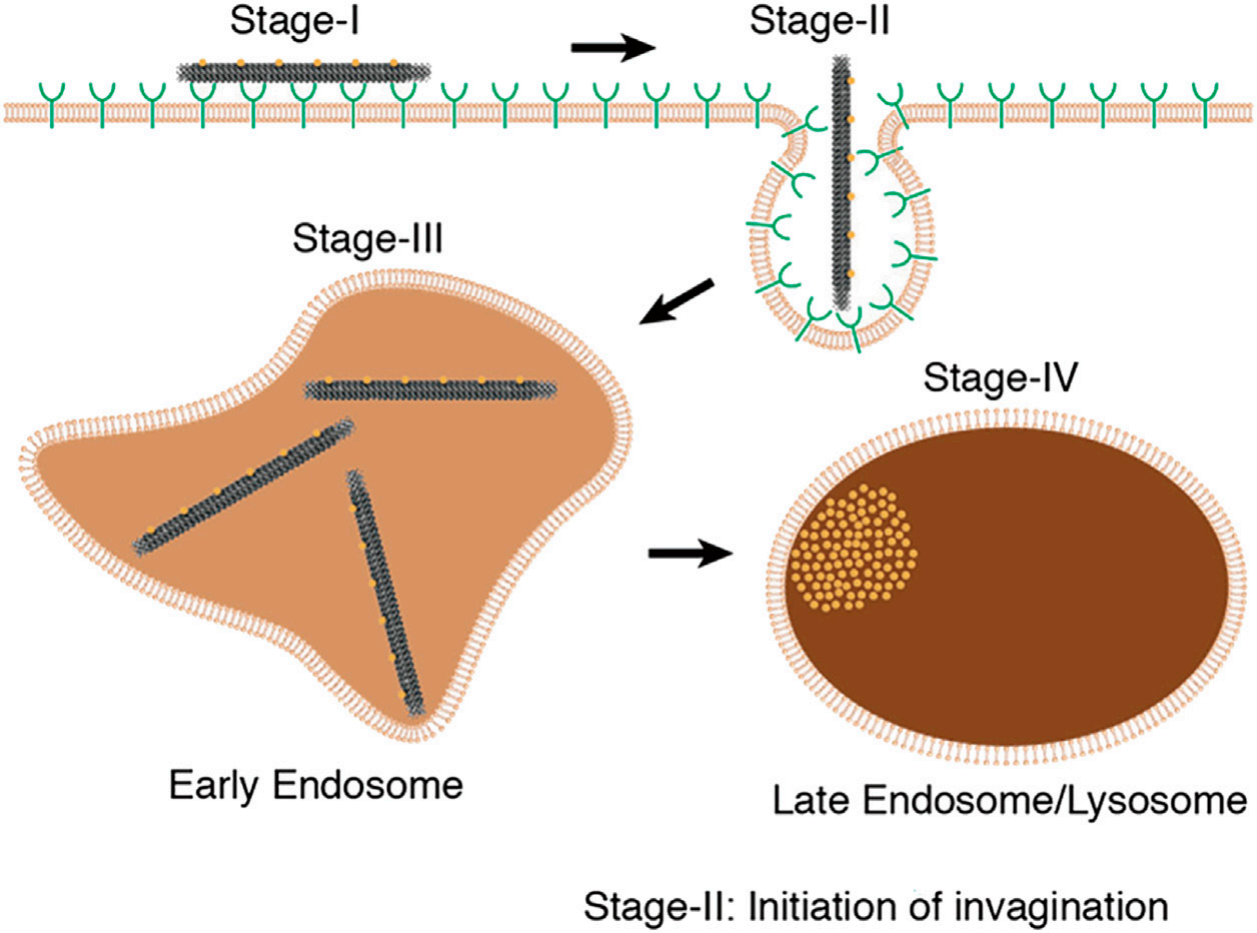
The internalization process of DONs includes four primary steps, including adsorption, endocytosis, early endosome formation, and late endosome formation. Reproduced with permission (Wang et al., 2018) Copyright © 2018, American Chemical Society.

As a result, DNA origami has become widely used in targeted drug delivery, effectively improving the efficiency and accuracy of drug delivery while also opening up new avenues for drug treatment research. Research has indicated that the process of DONs internalization by tumor cells involves the mechanism of cell membrane engulfment and the formation of endocytic vesicles. The internalization process of DONs includes four primary steps, adsorption, endocytosis, early endosome formation, and late endosome formation. During adsorption, DONs interact with scavenger receptors on the cell membrane surface, tightly adhering to the cell membrane. The cell membrane then undergoes stretching movements and wraps around DONs, forming an endocytic vesicle. Subsequently, the endocytic vesicle forms small vesicles on the cell membrane to create an early endosome, which fuses with the endoplasmic reticulum, transforming into a late endosome or lysosome, and then gets decomposed (Wang et al., 2018) (Figure 3). In fact, the uptake and intracellular localization mechanisms will be highly structure and coating dependent, different types of DONs, along with their distinct modifications, have a substantial impact on cellular uptake (Balakrishnan et al., 2019; Chiu et al., 2019). Currently, there is no consensus regarding the cellular uptake process of DONs, and further research is needed to shed light on this matter.

#### 3.1.1 Chemotherapy drugs

Chemotherapy is a widely used drug treatment method in cancer therapy, with drugs such as doxorubicin, daunorubicin, metal complexes, fluorouracil, vincristine, paclitaxel, and others commonly utilized. These small molecule drugs enter cells and interfere with the normal cell cycle, promoting cell death and killing cancer cells; however, they also cause multiple and severe side effects due to their broad action targets and strong cytotoxicity against both normal and cancer cells.

**FIGURE 4:**
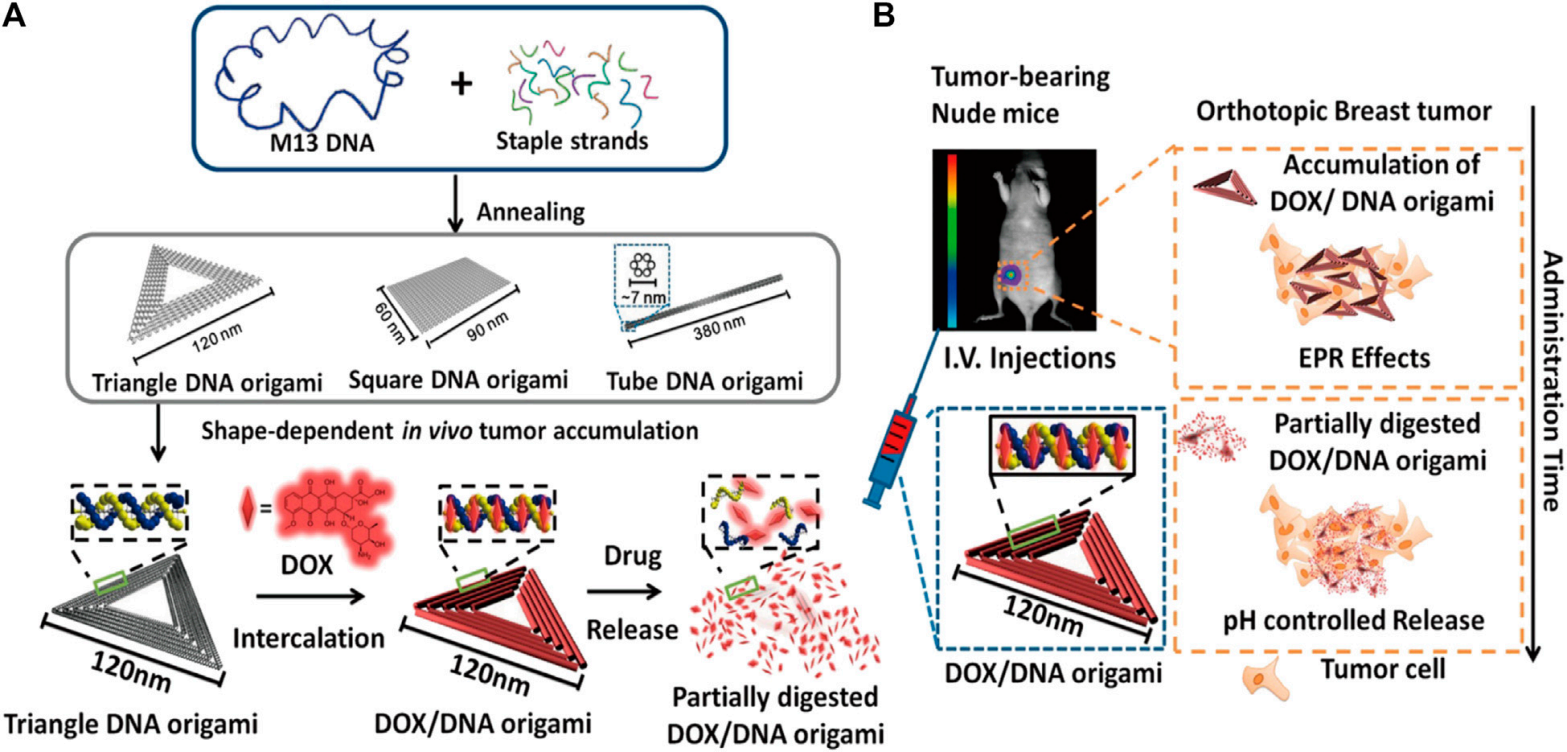
Diagram illustrating the use of triangular DONs for targeted delivery of DOX. Reproduced with permission (Zhang et al., 2014) Copyright © 2014, American Chemical Society. **(A)** Triangular, square, and tubular DONs were fabricated using DNA origami. Among them, triangular DONs exhibited the highest accumulation in tumors and were used for DOX loading. **(B)** Tail vein injection of DONs-DOX allowed them to enter the mouse breast tumor through blood circulation, accumulate in cancer cells, and eventually kill them.

DOX is a type of anthracycline drug, commonly used in chemotherapy. It achieves its therapeutic effect by primarily promoting cancer cell apoptosis through the inhibition of DNA synthesis. DOX has a strong affinity for binding to DNA and can be stably loaded onto DONs (Ijas et al., 2021). This composite structure of DONs-DOX exhibits high efficacy in targeting cancer cells and inducing stronger cytotoxicity than free DOX. Additionally, it promotes cancer cell apoptosis while limiting normal cell damage, reducing toxicity and side effects. Zhao et al. investigated the use of DONs-DOX to intervene in three different breast cancer cell lines, MDA-MB-231, MDA-MB-468, and MCF-7. The results showed that DONs-DOX exhibited higher cytotoxicity and a lower intracellular clearance rate compared to free DOX, thereby demonstrating its effectiveness in promoting the death of breast cancer cells (Zhao et al., 2012). Palazzolo et al. utilized compact short-tube DONs to load DOX liposomes and investigated their efficacy in breast cancer cell lines and mouse models. DONs-DOX exhibited high stability, biocompatibility, and improved anti-tumor efficacy and biological distribution of DOX in tumor-bearing mice. Additionally, it was observed to reduce bone marrow toxicity and evade immune system recognition (Palazzolo et al., 2019). Zhang et al. loaded DOX into triangular DONs and demonstrated significant anti-tumor efficacy in a mouse model of breast cancer without any observed systemic toxicity (Zhang et al., 2014) (Figure 4). Han et al. modified DONs with MUC1 adaptors to enhance the intracellular uptake efficiency of MCF-7 breast cancer cells while reducing the uptake efficiency of normal cells. DONs-DOX was found to demonstrate higher systemic safety and lower systemic toxicity compared to free DOX, making it more effective in inhibiting breast cancer cell proliferation (Han et al., 2018). Moreover, it exhibited significant cytotoxicity against conventional human breast cancer cells MCF-7 and doxorubicin-resistant cancer cells, inducing the reversal of the resistant phenotype and enhancing the cell-killing activity against doxorubicin-resistant MCF-7 cells (Jiang et al., 2012). Triple-negative breast cancer (TNBC) is a subtype of breast cancer that lacks expression of Estrogen Receptor (ER), Progesterone Receptor (PR), and Human Epidermal growth factor Receptor 2 (HER-2). Studies have shown that overexpression of FOLR1 plays an important role in the occurrence and development of TNBC (Necela et al., 2015; Song et al., 2016; Cheung et al., 2018). Pal et al. utilized Rothemund’s original triangle DNA origami to enable targeted delivery of DOX to FOLR1 overexpressing cells in TNBC. This approach reduced the side effects of DOX treatment while decreasing the required dose of DOX needed to kill cancer cells by approximately 31-fold (Pal and Rakshit, 2021). Unida et al. functionalized folate and connected the AS1411 aptamer to DONs loaded DOX, exhibiting more than a 231% increase in cytotoxicity against MDA-MB-51 cells compared to free doxorubicin. Additionally, they demonstrated that the bimodal nanocage loaded with doxorubicin helped overcome drug resistance by more efficiently entering triple-negative breast cancer cells (Unida et al., 2022). Furthermore, DOX often faces challenges such as rapid clearance and non-specific distribution, leading to off-target effects. To address this issue, prodrugs have been developed. They are a class of non-active agents that exert the pharmacological effects of the parent drug through site-specific biotransformation. The design of prodrugs targeting unique molecular defects in cancer cells (e.g., overexpression, mutation) is advantageous in improving pharmacokinetics and reducing off-target effects. However, many prodrugs involve systemic prodrug activation by non-specific stimuli (such as esterases, phosphatases, and lysosomal acidification), which may result in severe adverse reactions (Amly and Karaman, 2016). He et al. constructed rectangular DNA origami nanostructures of an amsacrine prodrug with tumor-targeting specificity enzyme (NQO1), which showed therapeutic selectivity towards NQO1-overexpressing MCF-7 cells and healthy L02 cells (He et al., 2023). This approach retained the characteristics of low clearance efficiency and high off-target effects of the amsacrine prodrug while having lower risk of side effects, demonstrating innovation and providing an effective strategy for precision cancer therapy. In addition to breast cancer, the DONs-DOX complex based on DNA origami technology has shown great potential in improving drug efficacy against colon cancer, prostate cancer, and ovarian cancer (Chang et al., 2011; Taghdisi et al., 2018; Zeng et al., 2018; Ge et al., 2020; Li et al., 2020; Yao et al., 2020; Yaghoobi et al., 2022; Singh and Deshmukh, 2023).

Metal complexes are coordination polymers formed by the coordination bond between a metal atom and ligands, exhibiting diverse composition and tunable structure. They have shown tremendous potential in anti-tumor applications. Some examples of metal complexes include Platinum (Pt), Ruthenium (Ru), Rhenium (Re), Osmium (Os), Rhodium (Rh), Copper (Cu), Nickel (Ni), Zinc (Zn), Titanium (Ti), etc (Sponer et al., 2006; Deubel and Chifotides, 2007; Galindo-Murillo et al., 2012; Alvarado-Soto and Ramirez-Tagle, 2015; Sayin and Ungordu, 2018; Haleel et al., 2019). Platinum-based anti-cancer drugs, including cisplatin, carboplatin, oxaliplatin, and other metal complexes, are widely used in cancer treatment. These drugs belong to a class of non-specific cell cycle drugs that form Pt-DNA adducts through hydration dissociation with the DNA structure after entering tumor cells. This mechanism leads to tumor cell death or apoptosis, thereby producing an anti-cancer effect. Researchers have designed a dodecahedral DNA nanocage connected by telomerase primers and telomere repeat sequences. Platinum nano-drugs were encapsulated in the dodecahedron, and when entering tumor cells, the telomerase enzyme precisely released the platinum nano-drugs from the cage, targeting and killing tumor cells while enhancing the therapeutic effect against cisplatin-resistant tumors and reducing toxicity to normal tissues (Ma et al., 2018). In recent years, research has shown that ruthenium complexes have ligand exchange kinetics similar to platinum-based anti-tumor drugs, low toxicity, and high selectivity, making them a very promising therapeutic agent with the potential to become a new class of metal complex anti-cancer drugs widely used in clinical practice (Brabec and Novakova, 2006; Thangavel et al., 2017). Researchers have used tetrahedral nanostructures to load Ru polypyridine complexes, which effectively enhanced the specific cellular uptake and cytotoxicity of HepG2 cells, induced ROS-mediated cell apoptosis, and in nude mouse models, the nano system specifically accumulated at the tumor site, reducing damage to the liver, kidney, lung, and heart functions of the mice (Huang et al., 2016).

In addition to DOX, other types of chemotherapy drugs have also been reported to be loaded onto DONs for drug delivery purposes. For instance, 5-fluorouracil (5-FU) and 5-fluoro-2′-deoxyuridine (FdUrd), which are effective against digestive tract cancer and other solid tumors, have been loaded onto DONs with attached cholesterol on the surface to enhance cellular uptake. Compared to traditional 5-FU and FdU, FdUrd-functionalized DNA nanomaterials display enhanced cell toxicity and a higher ability to induce apoptosis of colorectal cancer cells (Jorge et al., 2018). Similarly, mitoxantrone (MX), an anthracycline anti-cancer drug used in chemotherapy for hematological malignancies, has been loaded onto rod-shaped DONs to improve drug efficacy and overcome multi-drug resistance mediated by efflux pumps in leukemia cells at clinically relevant drug concentrations (Halley et al., 2016).

As mentioned earlier, traditional chemotherapy drugs cause unnecessary toxicity damage to healthy cells, and their dispersion in various tissues and organs through the bloodstream leads to problems such as insufficient drug efficacy, difficulty in concentration, and striping (Li et al., 2022). DONs manufactured using DNA origami technology can be customized and designed to produce highly specific targeted carriers against tumor cells, achieving precise drug delivery and localized targeted therapy. With molecular beacons on their surfaces, these highly specific DONs can bind to specific receptors on tumor cell surfaces, facilitating targeted recognition and drug delivery to the interior of tumor cells (Table 1).

**TABLE 1:** DNA origami in chemotherapy drugs delivery.

Drug	DONs	Modify	Function	References
DOX	nanotube DNA origami	-	promote apoptosis of human breast cancer cells	Zhao et al. (2012)
DOX	short tube DNA origami	-	improves the antitumoral efficacy, biodistribution of doxorubicin in tumor-bearing mice, decreases bone marrow toxicity	Palazzolo et al. (2019)
DOX	triangle-shaped DNA origami	-	Inhibition of breast cancer cell growth in mice	Zhang et al. (2014)
DOX	tetrahedral multivalent DNA nanocages	MUC1-aptamers	enhanced the intracellular uptake efficiency in MCF-7 tumor cells, reduce that of normal cells	Han et al. (2018)
DOX	triangular- and tubular-shaped DNA origami	-	enhancement of cell-killing activity to doxorubicin-resistant MCF 7 cells	Jiang et al. (2012)
DOX	triangular DNA origami	FOLR1-aptamers	targets and kills FOLR1 overexpressing cells	Cheung et al. (2018)
DOX	obtained DNA origami	folate and AS1411 aptamer	increase the cytotoxic effect on MDA-MB-231 cells	Pal and Rakshit (2021)
doxorubicin-derived prodrugs	rectangle DNA origami	NQO1	ncrease the cytotoxic effect on NQO1-overexpressing MCF-7 cells	He et al. (2023)
DOX	icosahedra DNA origami	MUC1-aptamers	efficient and specific internalization for killing epithelial cancer cells	Chang et al. (2011)
DOX	3D DNA triangle	-	enhanced cellular uptake capability	Zeng et al. (2018)
DOX	holliday junction DNA origami	AS1411-aptamers	enhanced the antitumor efficacy *in vivo* without boosting the adverse effects	Yao et al. (2020)
DOX	three-way junction pocket DNA nanostructure	AS1411-aptamers	reduce the cytotoxic effects of Dox against nontarget cells	Taghdisi et al. (2018)
DOX	DON of six helical bundles	2-[3-(1,3-dicarboxy propyl)-ureido] pentanedioic acid	selective delivery of Dox to the PSMA + cancer cell line LNCaP	Ge et al. (2020)
DOX	rectangle DNA origami	-	penetrate ovarian cancer cells efficiently	Li et al. (2020)
DOX	triangle-shaped DNA origami	-	specific delivery into tumor cells	Singh and Deshmukh (2023)
DOX	multi-storey DNA nanostructure	AS1411-aptamers	restrict tumour growth, increase survival rate, and accumulate significantly more in the tumour site than free DOX.	Yaghoobi et al. (2022)
cisplatin	DNA Icosahedron	Telomerase	enhanced anticancer efficacy in drug-resistant carcinoma, reduced toxicity to normal tissues	Sponer et al. (2006)
ruthenium polypyridyl complexes (RuPOP)	tetrahedral DON	-	enhances specific cellular uptake, drug retention and cytotoxicity against HepG2 cells	Huang et al. (2016)
FdUn	DNA tetrahedron and rectangle DNA origami	cholesterol	enhanced cytotoxicity and higher ability to trigger apoptosis in colorectal cancer cells	Jorge et al. (2018)
daunorubicin	rod-like DNA origami	-	enhanced drug efficacy	Halley et al. (2016)

#### 3.1.2 Nucleic acid drugs

Malignant tumors often result from multiple oncogenes’ activation and tumor suppressor genes’ inactivation. Nucleic acid drugs, such as CpG, siRNA, and antisense oligonucleotides (ASO), directly regulate the expression of specific genes associated with malignant tumor proteins at the genetic level. These drugs offer several advantages over traditional anti-tumor drugs, including simple sequence design, easy synthesis and modification, strong target specificity, a clear mechanism of action, a wide therapeutic range, and long-lasting efficacy. Consequently, they have become increasingly popular in anti-tumor treatment (Zou et al., 2017; Subhan and Torchilin, 2019; Wu et al., 2021b; Xu et al., 2021b; Meng et al., 2022; Noriega-Rivera et al., 2022; Revenko et al., 2022).

Nucleic acid drugs are composed of independent DNA or RNA sequences that have significant potential in clinical applications. However, their use is limited due to poor stability *in vivo*, low cellular uptake efficiency, and the potential for immune reactions (Hu et al., 2019). To overcome these limitations, nucleic acid drugs can be loaded onto DONs, providing numerous benefits (Roberts et al., 2020). The natural affinity between nucleic acid drugs and DNA makes them easy to load onto DONs. DONs-based nucleic acid drug delivery systems deliver several advantages, including protection from degradation by avoiding direct exposure to nucleases. Moreover, the outer layer of the DNA nanostructure effectively blocks premature degradation, enhancing their stability *in vivo* (Liu et al., 2012; Jiang et al., 2019). Modified DONs also improve the uptake efficiency of nucleic acid drugs in the target tissue, allowing accumulation in the surrounding tissues of the tumor (Kumar et al., 2016). Additionally, modification with peptides, aptamers, or antibodies enhances the specific binding of nucleic acid drugs to cancer cell receptors, thus improving their targeting ability (Lee et al., 2012). Lastly, the addition of DONs increases the molecular weight of nucleic acid drugs, making them less susceptible to rapid clearance (Lee et al., 2012; Kumar et al., 2016). This prolongs their half-life *in vivo* and allows for a prolonged therapeutic effect (Liu et al., 2012; Kumar et al., 2016). By overcoming the limitations of traditional nucleotide-based drugs, DONs-based nucleic acid drug delivery systems offer enormous promise in anti-tumor treatment and patient outcomes.

Cancer cells can evade recognition and attack by the immune system, which significantly contributes to their sustained survival and proliferation *in vivo*. CpG drugs containing non-methylated CpG as the core sequence provide an effective solution to this problem. These drugs contain multiple human immune-stimulating sequences and have strong immune-stimulating activity (Krieg, 2002; Chen et al., 2021). After entering immune cells, they promote the production of immune-related factors to clear immune-escaped cancer cells. Using DNA origami technology, various DONs such as tetrahedrons, multi-legged animals, DNA nanotubes, and wireframes can be fabricated, and CpG can be loaded onto these structures to enhance its resistance to degradation by nucleases in serum. This improves its ability to enter macrophages and dendritic cells. Once inside macrophages, CpG sequences are recognized by Toll-like receptors, and downstream signaling pathways induce immune stimulation. This promotes high levels of secretion of pro-inflammatory cytokines such as tumor necrosis factor-alpha (TNF-α), interleukin-7 (IL-7), and interleukin-12 (IL-12), strengthening the immune system’s surveillance and clearance of cancer cells (Rattanakiat et al., 2009; Li et al., 2011; Schuller et al., 2011; Mohri et al., 2012a; Mohri et al., 2012b; Ouyang et al., 2013; Ouyang et al., 2014; Qu et al., 2017; Du et al., 2022). To further enhance the immune response to cancer cells, Kang et al. designed a self-assembling nanoplatform using DNA origami technology that contains nitrosylated CD4^+^ T cell epitopes and CpG (Kang et al., 2023). This adjuvant CpG explicitly activates Toll-like receptor 9 and promotes the presentation of new antigens on dendritic cells, starting and prolonging new antigen-specific CD8 T cell responses, and significantly inhibiting tumor growth. Similarly, Comberlato et al. arranged active CpG motifs on a DNA origami disk at the nanoscale (Comberlato et al., 2022). When the CpG motif was located 7 nm away from Toll-like receptor 7, it could better combine with RAW 9.264 macrophages, achieving stronger immune activation. Using DNA origami technology to deliver CpG drugs, a promising strategy has been developed to stimulate the immune system to fight against cancer cells.

siRNA is a unique type of double-stranded RNA structure that can trigger the degradation of target sequences by binding completely and rendering the Argonaute-2 protein inactive. This process effectively inhibits the translation of specific genes, leading to the suppression of gene expression and modification of disease effects. One promising strategy for the targeted delivery of siRNA for cancer treatment involves using DONs as efficient delivery carriers, which can be customized through surface modifications to selectively target cancer cells and silence malignant tumor-specific protein-related genes (Lee et al., 2012; Chen et al., 2015). In earlier studies on DNA tetrahedron research, Han et al. utilized DNA tetrahedra as a foundation to construct a novel DNA-based nanogel for intracellular siRNA delivery. This approach successfully protected siRNA molecules and facilitated significant gene silencing upon cellular uptake. The developed nanogel exhibited potential applications in anti-tumor therapies and also provided valuable insights for utilizing DNA origami techniques in the delivery of nanostructures for siRNA delivery (Xue et al., 2019). The anti-apoptotic protein Bcl-2 plays a critical role in both cancer development and drug resistance (Zhang et al., 2021). To address this challenge, Rahman et al. developed rectangular and tubular DNA nanocarriers of varying sizes for delivering targeted siRNA against Bcl-2. Their studies demonstrated significant inhibition of tumor progression associated with Bcl-2 overexpression, both *in vitro* and *in vivo*. In summary, targeting Bcl-2-related genes using siRNA-loaded DONs represents a promising therapeutic strategy for treating tumors (Rahman et al., 2017).

**FIGURE 5:**
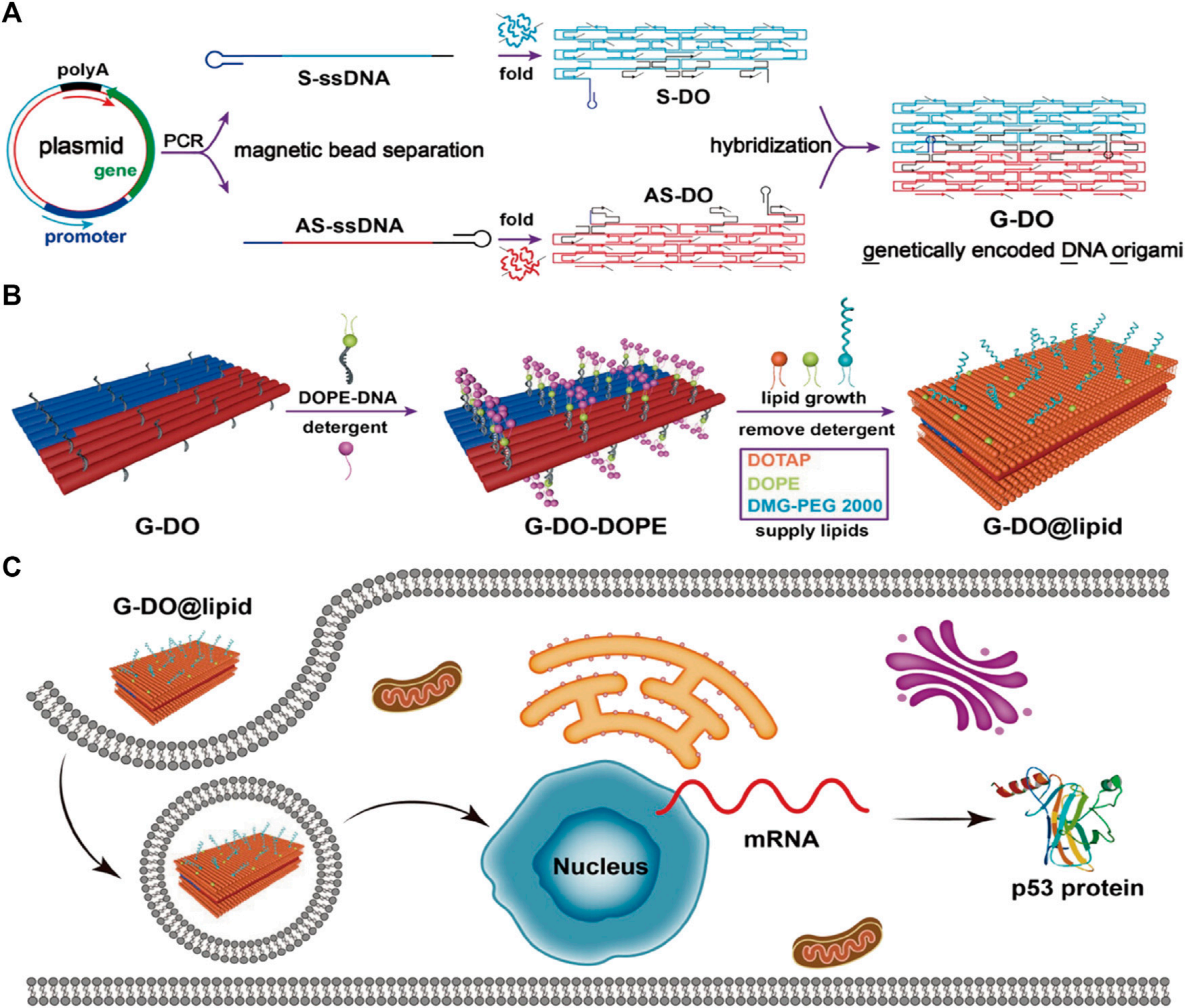
DNA origami is used to make nanostructures carrying gene drugs. Reproduced with permission (Wu et al., 2023) Copyright © 2023, American Chemical Society. **(A)** Folding the ASO encoding the anti-tumor gene (p53) and its complementary antisense nucleotide chain into a DNA origami structure. **(B)** Surface modification of the above-mentioned DONs. **(C)** The above-mentioned DONs enter the cell nucleus and upregulate p53 protein expression.

ASOs are single-stranded oligonucleotide molecules that can bind to complementary target mRNA through base-pairing principles in the presence of ribonuclease H1. This leads to inhibition of the expression of target genes and regulation of protein expression upon entering cells. Recent studies have demonstrated that ASOs hold great promise for inhibiting the development of ovarian cancer (Wang et al., 2022d), colorectal cancer, lung cancer (Shen et al., 2021), and hepatocellular carcinoma (Ma et al., 2022a) while also having potential applications in immune therapy for tumors (Revenko et al., 2022). Moreover, ASOs carried by DONs exhibit higher stability and safety (Long et al., 2021), and can deliver TGF-β1 mRNA-targeting ASOs to liver cells to combat hepatic fibrosis (Kim et al., 2022). The p53 tumor suppressor gene serves as a common tumor-suppressing gene, and its inactivation plays a crucial role in tumor formation. Mutated p53 helps with cancer proliferation and metastasis (Kim et al., 2019; Hu et al., 2021). To address this issue, Wu et al. developed an innovative approach to fold the ASO encoding the anti-tumor gene (p53) and its complementary antisense nucleotide chain into a DNA origami structure. This structure was then coated with a lipid envelope on its surface and modified with tumor-targeted groups. As a result, this structure could specifically target cancer cells, efficiently penetrate the cancer cell membrane, and express the ASO and complementary antisense nucleotide chain inside the cell. This led to a significant upregulation of p53 protein in tumor cells, promoting cancer cell apoptosis, and ultimately treating malignant tumors (Wu et al., 2023) (Figure 5) (Table 2).

**TABLE 2:** DNA origami in nucleic acid drugs delivery.

Drug	DON	Modify	Function	References
CpG	Y-shaped DON	-	enhance immune irritation	Rattanakiat et al. (2009)
CpG	dendrimer-like DNA nanostructures	TAT peptide	enhance immune irritation	Qu et al. (2017)
CpG	3D DNA tetrahedra	-	enhance immune irritation	Li et al. (2011)
CpG	polypod-like structured DNA	-	enhance immune irritation	Mohri et al. (2012a)
CpG	tetrapod-like structured DNA	-	enhance immune irritation	Mohri et al. (2012b)
CpG	DNA origami tubes	-	enhance immune irritation	Schuller et al. (2011)
CpG	3D polyhedral wireframe DNA origami	-	enhance immune irritation	Du et al. (2022)
CpG	DNA nanoribbon	-	promote TNF-α	Ouyang et al. (2013)
CpG	DNA nanoribbon	-	promote TNF-α	Ouyang et al. (2014)
CpG	DNA tetrahedron structure	CD4+T cell epitope	facilitate the effective activation of neoantigen-specific CD8 T cells	Kang et al. (2023)
CpG	DNA origami disk	-	Enhance immune irritation	Comberlato et al. (2022)
siRNA	DNA tetrahedral nanoparticles	folate molecules	Inhibit tumor related target genetic expression	Lee et al. (2012)
siRNA	periodic DNA nanoribbons	-	Targeted delivery into cancer cells	Chen et al. (2015)
siRNA	rectangular and tubular shapes DNA nanostructures	-	suppressed tumor growth in a xenograft model that specifically correlated with Bcl2 depletion	Rahman et al. (2017)
ASO	DNA origami by employing two complementary DNA strands	lipids	the antitumor gene (p53) encoded DNA origami can elicit a pronounced upregulation of the p53 protein in tumor cells	Wu et al. (2023)

#### 3.1.3 Peptides and protein drugs

Peptide and protein drugs have emerged as a major focus in anti-tumor therapy, encompassing various biochemical molecules such as antibodies, cytokines, receptor molecules, and enzymes. These drugs offer several advantages owing to their high specificity, multifunctionality, and good biocompatibility. Unlike nucleic acid drugs and chemotherapy drugs, peptide and protein drugs do not cause toxicity in normal cells or induce permanent or random changes in gene sequences. Despite these benefits, peptide and protein drugs also suffer from some limitations. Firstly, they exhibit poor stability both inside and outside the body, rendering them vulnerable to enzymatic degradation and other environmental factors, resulting in a short biological half-life. Secondly, peptides and proteins may elicit an immune response and pose a risk of allergic reactions. Thirdly, a significant first-pass effect occurs in the liver and gastrointestinal tract, leading to low effective concentrations within the body. Fourthly, most proteins have large relative molecular weights, complex structures, and poor membrane penetration under neutral pH conditions, resulting in low bioavailability. However, DNA origami offers a promising solution to overcome these limitations. By preparing DONs, researchers can achieve breakthroughs in maintaining the biological activity, targeting, controlled release, and prolonged duration of action of peptide and protein drugs.

Peptides and proteins have been precisely and conveniently assembled onto DONs (Zhou et al., 2018; Brown et al., 2022). To enhance the targeting of DON-protein structures, researchers have suggested using DONs and zinc fingers (ZnFs) to assemble targeted protein delivery nanoparticles (Ryu et al., 2018). Y-shaped DNA origami modified with ZnFs can deliver PTEN tumor suppressor proteins into cells, regulating kinase signaling pathways and inhibiting cancer cell growth (Ryu et al., 2020). Tumor necrosis factor (TNF) is involved in regulating the cell cycle of tumor cells. DONs modified with TNF-related apoptosis-inducing ligands can promote apoptosis of human breast cancer cells, providing a new strategy for ineffective TNF superfamily activation methods based on ligands and antibodies (Wang et al., 2021a). Ma et al. utilized flat rectangular DONs loaded with tumor necrosis factor-related apoptosis-inducing ligands (TRAIL) trimer to induce apoptosis of tumor cells. Ribonuclease (RNase) A is an RNA hydrolyzing enzyme (Ma et al., 2023). Zhao et al. added RNaseA and MUC1 adaptors to rectangular DNA origami nano-sheets, enabling DONs to specifically enter tumor cells and hydrolyze RNA inside cancer cells, inhibiting tumor progression. Caspase-9 is a pro-apoptotic protein (Zhao et al., 2019). Rosier et al. combined it with DONs to enhance the regulation of cell apoptosis. Additionally, overexpression of epidermal growth factor (EGF) and adrenaline receptors (Eph) promotes cancer development (Rosier et al., 2020). By rearranging the distribution and proportional quantity of corresponding binding ligands on the surface of DONs, cancer cell transcription and invasion can be effectively inhibited (Huang et al., 2019; Verheyen et al., 2020). Berger et al. arranged Fas ligands on DNA origami sheets into hexagonal arrays with a molecular spacing of 10 nm, revealing their high capability to induce cellular apoptosis. This finding holds great promise for applications in the field of tumor therapy (Berger et al., 2021). Wang et al. developed a DNA origami template-adaptor nanoarray (DOTA) that enables precise programming of receptor tyrosine kinase (RTK) oligomerization at the ligand-receptor interface. The DOTA chip allows for precise control over the activation levels of RTK signaling by defining valency, distribution, and stoichiometry of the receptor tyrosine kinases. This manipulation promotes the transition from epithelial to mesenchymal-like cells and regulates cellular behavior (Wang et al., 2022a).

One effective approach for treating tumors involves inducing thrombus formation to obstruct the tumor’s blood supply by activating thrombin activity (Li et al., 2019b). However, thrombin is a non-specific agent, and its release outside of tumor vessels can result in systemic thrombosis, which may lead to serious conditions such as pulmonary embolism, myocardial infarction, cerebral infarction, or even pose a risk to life. Nevertheless, the development of DNA nanorobots utilizing DNA origami provides a promising solution for the targeted delivery of thrombin. These DNA nanorobots can carry thrombin and release it solely within tumor-related blood vessels upon detecting specific tumor-related proteins. This targeted delivery method ensures that thrombin only induces thrombus formation where needed, effectively suppressing tumor growth while reducing the potential risks associated with systemic thrombosis (Li et al., 2018; Li et al., 2019a; Singh and Deshmukh, 2023).

**FIGURE 6:**
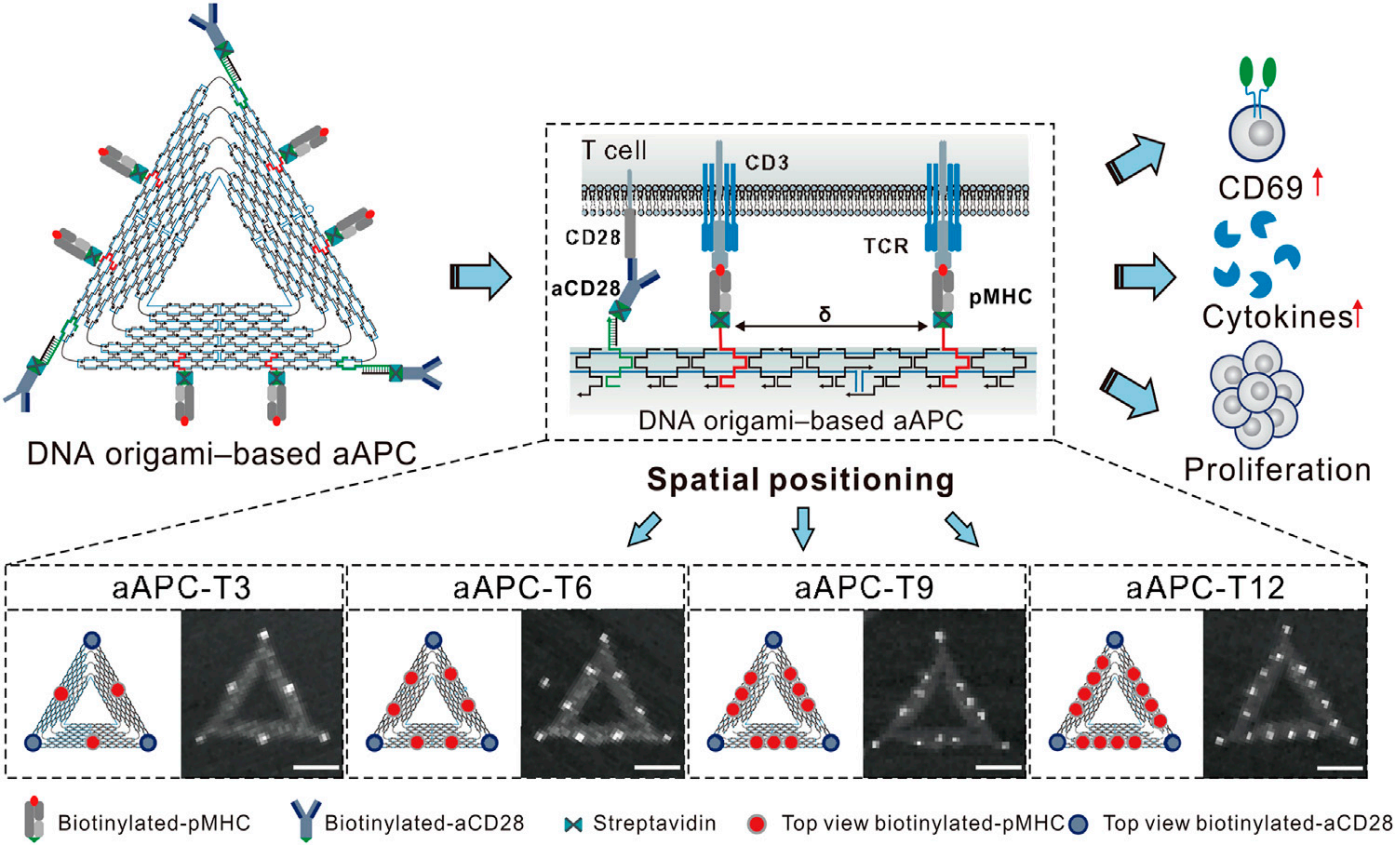
The aAPCs designed based on DNA origami were used to regulate T cell activation. Reproduced with permission (Sun et al., 2022) under copyright Creative Commons Attribution 4.0 International License (CC-BY license).

Immunotherapy, which utilizes the body’s immune system, is a major focus of cancer treatment research. DNA origami holds immense potential in the field of immunotherapy by enhancing immune stimulation activity and improving targeting (Ouyang et al., 2017). Additionally, DNA origami plays a critical role in adoptive immunotherapy and cancer vaccines. Sun et al. optimized DNA origami structures for use in nanoscale artificial antigen-presenting cells (aAPCs), which have efficient signal delivery and great potential in adoptive cell therapy. By anchoring co-stimulatory ligands that target CD28 antibodies and T cell receptor (TCR) ligand peptide-major histocompatibility complex (pMHC) on its three vertices and three edges, their DNA-origami-based aAPCs demonstrated higher tumor growth inhibition in adoptive immunotherapy (Sun et al., 2022) (Figure 6). In another study, Liu et al. assembled molecular adjuvants and antigen peptides into the lumen of tubular DNA nanostructures (Liu et al., 2021). These structures were activated in subcellular environments to trigger T cell activation and cancer cell toxicity, which not only reduced tumor size but also prevented recurrence. This approach presents a promising avenue for the development of cancer vaccines. Kern et al. developed a DNA-origami-based phagocytic system carrying FcγR receptor ligands, which enhanced macrophage phagocytosis by regulating these ligands’ density on the DNA origami structure (Kern et al., 2021). This has significant implications for the design of immunotherapies, as macrophages play a crucial role in clearing pathogens and diseased cells through Fcγ receptors (FcγRs) driven regulation. Moreover, PD-1 is an inhibitory receptor expressed on activated T cells that suppresses T cell signaling and effector function, making it a current research hotspot in cancer immunotherapy. FANG et al. found that DNA origami plates modified with CD3 and CD28 activating antibodies (FS-α-CD3-CD28) induced strong T cell activation. The spatial organization of PD-L1 determines its ability to regulate T cell signaling, and this finding may guide the development of future nanomedicine-based immune modulation therapies (Fang et al., 2021) (Table 3).

**TABLE 3:** DNA origami in peptides and protein drugs delivery.

Drug	DON	Modify	Function	References
PTEN tumour suppressor protein	Y-shaped DNA origami	ZnFs	inhibit cancer cell growth	Ryu et al. (2018)
TNF-related apoptosis-inducing ligand-mimicking peptides	hexagonally DON	-	promote breast cancer cell apoptosis	Ryu et al. (2020)
tumor necrosis factor related apoptosis inducing ligand	flat rectangular DNA origami	-	Promote apoptosis	Ma et al. (2023)
RNaseA	rectangular DNA origami nanosheets	MUC1 aptamer	targeted kill tumor cells	Zhao et al. (2019)
caspase-9	DNA origami platform	-	regulate apoptosis	Rosier et al. (2020)
EGF-binding ligands	triangular DNA	A20FMDV2	targeting human skin melanoma cells and	Huang et al. (2019)
thrombin	tube-like	-	proceeding to tumor necrosis and tumor growth inhibition	Li et al. (2020)
thrombin	tube-like	binds nucleolin	tumor necrosis and inhibit tumor growth	Li et al. (2018)
thrombin	tube-like	binds nucleolin	tumor necrosis and inhibit tumor growth	Li et al. (2019a)
IgG antibody	rectangular DNA origami structure	-	increased control over the orientation of antibodies in nanostructures	Ouyang et al. (2017)
TCR ligands peptide ligands anti-CD28 antibody	triangle-shaped DNA origami	-	inhibit tumor growth	Sun et al. (2022)
tumour antigen peptide; CpG loops; dsRNA	tubular DNA nanostructure	-	tumour regression in mouse cancer models	Liu et al. (2021)
Fcγ-ligand	DNA origami pegboards	-	enhances engulfment, increase efficiency of the engulfment-initiation process	Kern et al. (2021)
FS-α-CD3-CD28; PD-L1 ligands	DNA origami flat	-	inhibit T Cell activation *in vitro*	Fang et al. (2021)

### 3.2 Multifunctional drug delivery system

A drug delivery system is considered multi-functional if it can perform multiple functions. DNA origami, due to its highly customizable nature, can be utilized to create carriers that can contain numerous drugs with different therapeutic effects. This enables a comprehensive treatment approach encompassing various methods such as photoacoustic diagnosis, photothermal therapy, chemotherapy, immunotherapy, gene therapy, and more. Hence, DNA origami is an essential component in the advancement of multi-functional delivery systems. Recently, several successful attempts have been made to utilize DNA origami in the production of multi-functional drug delivery carriers.

Drug therapy is a crucial approach to treating cancer. However, the problem of drug resistance has become increasingly common among cancer patients as they undergo treatment over extended periods. This resistance limits the cytotoxic effect of anti-cancer drugs on cancer cells, making it one of the primary reasons for the failure of cancer treatment. Consequently, overcoming cancer cell resistance is a significant challenge in modern clinical work. Gold nanomaterials (AuNPs) have unique photoelectric, physical and chemical properties, and excellent biocompatibility, making them an attractive option in current cancer treatments (Boisselier and Astruc, 2009). They are frequently utilized as drug carriers to deliver drugs into tumor cells and kill cancer cells through photothermal effects (Alle et al., 2022). A new method of treating resistant tumors involves loading gold nanorods and drugs onto DNA origami nanostructures (DONs) for targeted delivery to resistant tumor cells. By combining chemotherapy drugs and targeted drugs, they can work together to kill resistant tumor cells (Kong et al., 2016; Song et al., 2017; Zhang et al., 2017; Jin et al., 2019). To address deeper cancer cells in tumor tissues, Gu et al. combined DOX, gold nanoparticles (GNP), and tetrahedral DNA nanostructures (TDNs) (Gu et al., 2022). The structure was designed to cause damage to tumor tissue at different depths based on changes in pH value. Initially, the overall nanostructure accumulates in tumor cells and works together. When the pH value of the tumor microenvironment is around 6.5, the TDN transforms from a double helix into a triple structure through DNA sequence connection, separates from GNP, penetrates deep into the tumor stroma, and internalizes into cells using GNP photothermal therapy to kill the tumor. Finally, in the acidic lysosome with a pH of 5.0, TDN releases DOX by forming an i-motif structure to kill deep cancer cells using DOX toxicity.

**FIGURE 7:**
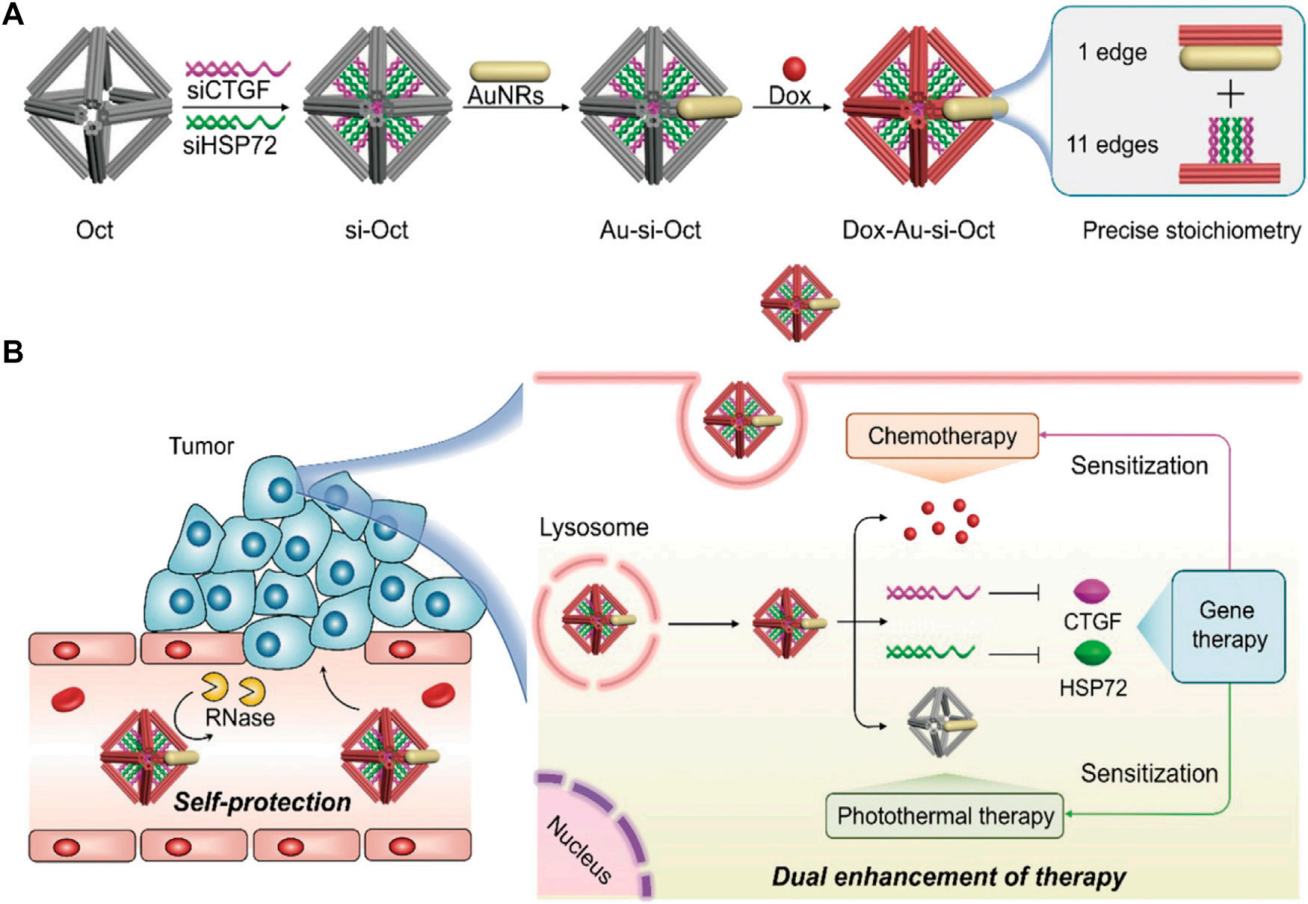
Octahedral DNA nanostructures fabricated by DNA origami are utilized for multifunctional drug delivery. Reproduced with permission (Xu et al., 2021a) Copyright © 2021, John Wiley and Sons. **(A)** The siRNA (siCTGF and siHSP72), DOX, and AuNRs were jointly mounted on the octahedral DONs. **(B)** This composite structure maintains stability in the bloodstream and exerts chemotherapy, photothermal therapy, and gene therapy upon internalization by cancer cells.

DONs, in addition to gold nanoparticles, have the potential to treat drug-resistant tumors when used in combination with chemotherapy drugs and nucleic acid drugs. Co-delivery of Dox and two different ASOs using DONs by Pan et al. effectively inhibited the expression of drug-resistant proteins B-cell lymphoma 2 (Bcl2) and P-glycoprotein (P-gp), thus enhancing the therapeutic effect on chemoresistant cells (Pan et al., 2020). In another study, Xu et al. loaded siRNA, DOX, and AuNRs onto octahedral DONs as carriers, downregulating the expression of connective tissue growth factor (CTGF) and heat shock protein 72 (HSP72) in drug-resistant cancer cells. This made cancer cells sensitive to both chemical drugs and thermal therapy (Xu et al., 2021a) (Figure 7). The multifunctional DONs constructed by Liu et al. delivered siRNA and DOX into MCF-7R cells through active targeting and controlled release, synergistically inhibiting tumor growth without significant systemic toxicity (Liu et al., 2018a). Wang et al. also used DONs to deliver siRNA and DOX, which effectively enhanced cell toxicity and inhibited tumor growth without causing systemic toxicity (Wang et al., 2021b). Additionally, Wu et al. embedded platinum-based drug 56MESS and anti-EGFR nanobody into DNA tetrahedral structures (Wu et al., 2019). This combination medication targeted and killed cancer cells with platinum-based drugs while blocking EGFR-related signal transduction in cells with high EGFR expression, achieving multi-drug combination therapy.

To summarize, the multifunctional drug delivery system that is founded on DONs can trigger synergistic effects of physical, chemical, genetic, immune, and other treatments in a targeted manner toward cancer cells. This approach leads to enhanced efficacy in killing drug-resistant cancer cells. As a result, the DNA origami-based multifunctional drug delivery system presents a promising novel strategy for treating cancers that are resistant to multiple drugs (Table 4).

**TABLE 4:** DNA origami in multifunctional drug delivery system.

Drug	DON	Modify	Function	References
DOX, Gold nanorods	triangular shaped DNA origami structures	MUC-1aptamer	the P-glycoprotein (multi-drug resistance pump) expression of multi-drug resistant MCF-7 (MCF-7/ADR) cells was downregulated	Song et al. (2017)
DOX, Gold nanorods, antibody, Erlotinib or Afatinib	2D double-stranded DNA	-	formed with great therapeutics loading capacity for different cancer treatments with synergism and multi-drug resistance inhibition	Kong et al. (2016)
	3D rectangle shape DNA origami			
gold nanorods, molecular targeted drugs	rectangular shaped DNA origami	phospholipid; pH responsive calcium phosphate [Ca3 (PO4)2 ] nanoparticles	induces cancer cell apoptosis, reduces the multi-drug resistance, and enhances the targeted killing efficiency towards human epidermal growth factor receptor 2 positive cells	Zhang et al. (2017)
DOX, Gold nanoparticle	DNA nanorobots	polydopamine	exhibits an *in vitro* synergetic antitumor effect of photothermal therapy and chemotherapy and outstanding tumor-targeting efficiency in both *in vitro* and *in vivo* environments	Jin et al. (2019)
DOX, gold nanoparticle	tetrahedral DNA nanostructure	-	effective deep penetration into the tumor core with good antitumor efficacy and satisfactory biocompatibility	Gu et al. (2022)
DOX, ASOs	DNA origami platform	MUC1 aptamers	circumvent multi-drug resistance and significantly enhance cancer therapy in Hela/ADR and MCF-7/ADR cells	Pan et al. (2020)
siRNA, DOX, AuNRs	octahedral DNA origami frameworks	-	achieving effective downregulation of connective tissue growth factor (CTGF) and heat shock protein 72 (HSP72) for dual sensitization of cancer cells to chemodrugs and hyperthermia	Xu et al. (2021a)
RNAi, DOX	triangular DNA Origami	MUC1 aptamer	synergistically inhibit tumor growth without apparent systemic toxicity	Liu et al. (2018a)
siRNA, DOX	tubular DNA Nanodevice	trans-activator of transcription (TAT) peptide	induced potent cytotoxicity and tumor growth inhibition	Wang et al. (2021b)
56MESS, EGFR nanobody	double-bundle DNA tetrahedron	-	exhibit selectivity for cells with elevated EGFR expression and combined tumor therapy without obvious systemic toxicity	Wu et al. (2019)

## 4 Challenges and prospects

DNA origami has made significant strides in drug delivery applications. However, there are still some obstacles to overcome. Firstly, the stability of DONs within the human body is not yet optimal and can be affected by several factors such as enzymes, pH levels, electrolytes, and temperature (Bila et al., 2019). The ability to stably load drugs into the body is critical for a drug delivery platform, as it significantly affects the safe entry of loaded drugs into targeted cells. Due to the fact that physiological fluids and blood serve as the primary media for the *in vivo* transportation of DONs, it is necessary to further enhance the stability of DNA origami structures within the circulatory system. This will enable them to effectively deliver anti-tumor drugs and fulfill their therapeutic potential. Existing research findings suggest that adding coatings to DNA nanostructures, such as lipids (Perrault and Shih, 2014), nucleotides (Kim and Yin, 2020), peptides (Auvinen et al., 2017), proteins (Wang et al., 2020), and other materials, can effectively enhance the *in vivo* stability of DONs. In the future, with the discovery and utilization of more valuable coatings, the stability of DONs is expected to be further strengthened. Xin et al. discovered that the use of cryoprotectants such as glycerol and trehalose effectively prevents the damage to DONs during multiple freeze-thaw cycles, ensuring their long-term preservation for drug *in vitro* applications (Xin et al., 2020). In contrast, Roodhuize et al. investigated the binding mechanisms between various ions and DONs due to the instability and degradation caused by low concentrations of cations in physiological fluids (Roodhuizen et al., 2019). This study lays the foundation for improving the stability of DONs in the liquid environment inside the body.

Modifying the surface of DONs can enable targeted delivery of anti-tumor drugs. However, off-target effects are still observed, which reduce the specific distribution of anti-tumor drugs in tissues, lower delivery efficiency, and pose a risk of severe adverse reactions. Therefore, improving the specific distribution capability of DONs in tissues and reducing off-target effects remains a crucial area of research in the field of DNA origami. One effective strategy to enhance target-specific binding affinity is to spatially arrange ligands on DONs, maximizing their binding with target proteins (Kwon et al., 2020). Additionally, increasing the number of ligands on DONs’ surfaces can also strengthen their targeting ability. For example, Lacroix et al. decorated DNA nanotubes with varying quantities of amphiphilic dendritic DNA (DDNA) to enhance the binding activity of DNA nanostructures with receptors, which were subsequently used for siRNA delivery (Seeman and Sleiman, 2017). In order to achieve more specific drug delivery, DONs need to exhibit more intelligent responsiveness to the external environment. In intelligent drug delivery system, Scherf et al. developed a DNA origami nanocage that responds to external stimuli (Scherf et al., 2022). The nanocage has small molecules on its surface that can be triggered to open and release drug payloads at specific sites upon receiving particular external cues. Additionally, even though DONs display high biocompatibility and low toxicity, they may still trigger an immune response if rejected by the body’s immune system (Surana et al., 2015). As drug delivery systems continue to evolve, precision and intelligence have become increasingly important.

Equally important is achieving efficient cellular uptake and release of DONs. Due to the negative charge and hydrophilic nature of DONs, passive transport into cells becomes challenging. Endocytosis is the primary mechanism by which DONs are internalized by cells, with clathrin-mediated endocytosis, caveolin-independent endocytosis, and macropinocytosis being significant pathways (Lim et al., 2015; Vindigni et al., 2016). Constructing more suitable shapes of DONs can facilitate cellular endocytosis. For instance, DNA nanostructures with a high aspect ratio are more readily absorbed by cells (Ouyang et al., 2013). Larger-sized DNA nanostructures exhibit higher cellular uptake efficiency compared to smaller ones. Additionally, rod-shaped DNA nanostructures perform better than similarly sized tetrahedral DNA nanostructures (Wang et al., 2018). Modifying DONs with folate, ligands, proteins, and other molecules can also enhance cellular internalization. Research has shown that viral capsid proteins can be electrostatically interacted with DONs, leading to their binding and self-assembly. Compared to bare DONs, the ability of virus protein-coated DONs to be internalized into cells was found to be increased by 13-fold (Mikkila et al., 2014). Furthermore, only those drugs that interact with DNA, such as CpG and siRNA, can be encapsulated or conjugated to these DONs. For other drugs, they may need to be combined with DNA, which could introduce additional barriers to their release within cells. For example, in the case of DOX, both pH values and DNA origami shapes can affect its release efficiency (Zhao et al., 2012; Zhang et al., 2014). Designing DONs that are compatible with these drugs is essential for their efficient release. For instance, pH-sensitive DONs that are highly sensitive to the intracellular pH environment. Researchers have designed an octahedral DNA origami that senses pH value changes and can switch between simple cubic (SC) and simple tetragonal (ST) structures (Wang et al., 2022c). This scientific advancement holds promise for delivering drugs based on pH value changes. In the future, extensive research is needed to explore and discover suitable DNA origami shapes for efficient delivery of various types of drugs. This will enable enhanced drug release within cells, mitigating the adverse effects associated with these limitations.

In addtion, DNA origami holds great potential for application in tumor diagnosis, with capabilities to detect tumor markers and perform molecular imaging of tumors. Tumor cells express specific markers on their membranes, which can be identified using DNA origami. By utilizing the controllability and programmability of DNA, DNA origami allows for the design of DNA molecules that can specifically recognize these markers, resulting in highly sensitive detection of tumor markers or tumor mutation genes (Liu et al., 2015; Chen et al., 2022; Domljanovic et al., 2022; Palankar, 2022; Chen et al., 2023). Moreover, on DONs, tracer molecules such as fluorescent dyes (commonly cyanine dyes like Cy5.5), quantum dots (QDs), radioactive isotopes, and other imaging agents can be precisely arranged at the nanoscale (Zeng et al., 2021). Subsequently, functionalization of DONs with ligands and other molecules can enable their targeting to tumor-associated cells, facilitating superior imaging of tumor tissues (Meng et al., 2016). This technology boasts higher spatial resolution and better biocompatibility compared to traditional imaging techniques (Bhatia et al., 2011; Zeng et al., 2021; Zeng et al., 2022). Overall, DNA origami offers numerous benefits in tumor diagnosis, including improved early diagnosis rates and high-sensitivity, high-resolution imaging of tumor tissues. These advantages bring about new possibilities for tumor research and treatment.

In summary, DNA origami is advancing towards better stability, safety, and intelligence. It has the potential to revolutionize drug delivery in the future.

## 5 Summary

DNA origami is a novel self-assembly technique with unique programmability, addressability, and biocompatibility. This technology offers several advantages in drug delivery. First, it allows for precise control of drug delivery by designing DONs. This improves therapeutic efficacy while reducing side effects. Second, DONs have demonstrated the ability to effectively cross biological barriers, such as cell membranes and the blood-brain barrier, resulting in efficient drug delivery. Third, DNA origami can be customized according to the characteristics of different drugs and diseases, enabling personalized treatment. Fourth, DNA is a natural material in the body and has excellent biocompatibility, with no significant immune response or toxicity in humans.

However, there are some shortcomings of DNA origami that need further research. Firstly, preparing DONs through DNA origami is a complicated process requiring strict control of experimental conditions, which can lead to structural instability and uneven assembly. Secondly, large-scale production of DNA origami is still in its early stages, with high production costs, low efficiency, and batch-to-batch variability, which limits its promotion and application in practical use (Praetorius et al., 2017). Thirdly, DONs are susceptible to external factors such as pH, temperature, and ion concentration, which may cause structural instability and deactivation, affecting their drug delivery effect. Finally, the complex nucleic acid sequences in DONs may pose risks such as instability and tumor induction, requiring further research on their biosafety.

Despite these challenges, DNA origami has made significant progress in chemotherapy drugs, nucleic acid drugs, peptide and protein drugs, immunotherapy, and other areas, demonstrating great potential for application. Moreover, multifunctional drug delivery systems can be created by loading multiple drugs into DONs, which can exert a combined therapeutic effect and improve tumor resistance. Although further research is required to address the stability and safety concerns associated with DNA origami, existing research results indicate that it is expected to play an extensive role in drug delivery.
